# Osteoporosis, jawbones and periodontal disease

**DOI:** 10.4317/medoral.18298

**Published:** 2012-12-10

**Authors:** Rosario Guiglia, Olga Di-Fede, Lucio Lo-Russo, Delia Sprini, Giovan B. Rini, Giuseppina Campisi

**Affiliations:** 1 DDS, PhD Department of Surgical and Oncological Disciplines, Section Oral Medicine V. Margiotta, University of Palermo, Via del Vespro 129, 90127 Palermo, Italy; 2 DDS, PhD Department of Surgical and Oncological Disciplines, Section Oral Medicine V. Margiotta, University of Palermo, Via del Vespro 129, 90127 Palermo, Italy; 3 DDS, PhD Department of Surgical Sciences, University of Foggia, Viale L. Pinto 1,71100 Foggia, Italy; 4 MD, MS Department of Clinical Medicine and Emerging Diseases, University of Palermo, Via del Vespro 143, 90127 Palermo, Italy; 5 MD, MS Department of Clinical Medicine and Emerging Diseases, University of Palermo, Via del Vespro 143, 90127 Palermo, Italy; 6 DDS, MS Department of Surgical and Oncological Disciplines, Section Oral Medicine V. Margiotta, University of Palermo, Via del Vespro 129, 90127 Palermo, Italy

## Abstract

The association between osteoporosis and jawbones remains an argument of debate. Both osteoporosis and periodontal diseases are bone resorptive diseases; it has been hypothesized that osteoporosis could be a risk factor for the progression of periodontal disease and vice versa. Hypothetical models linking the two conditions exist: in particular, it is supposed that the osteoporosis-related bone mass density reduction may accelerate alveolar bone resorption caused by periodontitis, resulting in a facilitated periodontal bacteria invasion. Invading bacteria, in turn, may alter the normal homeostasis of bone tissue, increasing osteoclastic activity and reducing local and systemic bone density by both direct effects (release of toxins) and/or indirect mechanisms (release of inflammatory mediators). Current evidence provides conflicting results due to potential biases related to study design, samples size and endpoints. The aim of this article is to review and summarize the published literature on the associations between osteoporosis and different oral conditions such as bone loss in the jaws, periodontal diseases, and tooth loss. Further well-controlled studies are needed to better elucidate the inter-relationship between systemic and oral bone loss and to clarify whether dentists could usefully provide early warning for osteoporosis risk.

** Key words:**Osteoporosis, periodontitis, oral bone loss, tooth loss, edentulism, bone mineral density.

## Introduction

Osteoporosis (OP) is a systemic skeletal disease characterized by low bone mass and micro architectural deterioration of bone tissue, with a consequent increase in fragility and susceptibility to fracture of bones. In the past, OP was considered a physiological process associated with ageing, but today it is recognized as a multifactorial chronic systemic disease. OP may affect also the jawbones, whose structure may be impaired by other conditions resulting in bone loss. One of these, is periodontitis (PD), a chronic infection-mediated condition modulated by different genetic and environmental factors, characterized, in advanced forms, by loss of the soft tissue attachment to teeth and resorption of alveolar bone . PD is the prototype of a low grade local infection (bacteria of the oral plaque) associated with local (within the periodontal tissues) immune-inflammatory response causing periodontal tissue damages/destruction and a mild individual systemic inflammatory response contributing to the global inflammatory burden and to its related dangerous effects. PD is very prevalent in the general population in the same age range affected by OP. In fact, moderate and advanced periodontitis affects, respectively, approximately 30% and 10% of the adult populations of United States. Deep periodontal pockets (the clinical sign of periodontal attachment loss) are present in 2-18% of adults in western countries and at higher prevalence in developing countries (1) .

It might be expected that the alveolar bone destruction seen in periodontitis could be magnified in the presence of generalized skeletal disturbances such as OP. Nonetheless, it is increasingly becoming evident that PD may have several systemic implications (e.g. increased risk for cardiovascular disease), and hypothetical models exist linking OP and PD (1). It is well known that se-veral systemic and local factors may modulate the loss of bone mass and that some of them, as well as many risk factors ( Table 1), may be shared between the two conditions.

**Table 1:**
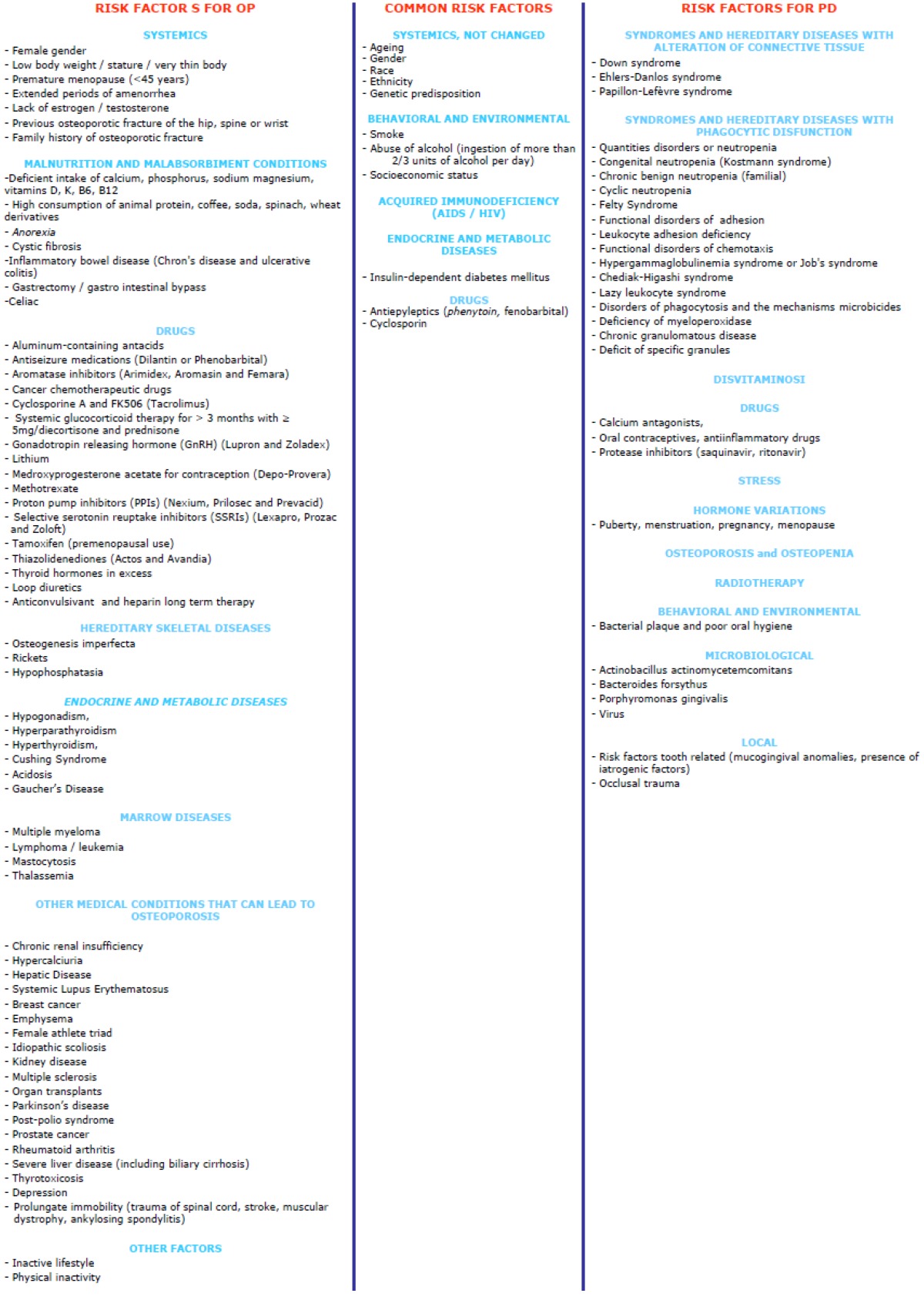
Risk factors for osteoporosis (OP) and periodontal disease (PD).

## Material and Methods 

The authors performed a literature review in MEDLINE/ PubMed and Cochrane Oral Health Group`s Trial Register by the main key words related (osteoporosis AND periodontitis, osteoporosis AND oral bone loss, osteoporosis AND tooth loss, osteoporosis AND edentulism, osteoporosis AND bone mineral density). In this paper we are going to review further evidence regarding the potential correlation between OP and PD.

## Results

-Osteoporosis and The Jawbones

OP can affect several skeletal sites including jawbones. Nonetheless, jawbones have some peculiar features related to the type of ossification and the high turnover induced by masticator mechanical stresses. Morphological studies have shown that the cortical bone porosity of the upper jaw increases with age; in addition, a considerable variation of the thickness and cortical porosity exists in different areas of the mandible (area of incisors, premolars and molars) in relation to sex, with significantly higher values in males than females. The body of the mandible and the posterior alveolar processes, consisting predominantly of cortical bone, are very similar to the diaphysis of long bones, while in the anterior alveolar processes of the mandible and in the alveolar processes of the jaw, bone architecture is mostly trabecular. According to some authors the rate of bone turnover at the level of alveolar processes would be greater than in long bones, so the loss of bone mass could manifest earlier at the alveolus than at other skeletal segments, thus, representing an early indicator of OP. These observations are consistent with those described by von Wowern et al. (2) who proposed that the mandible suffers from continuous modifications of bone mineral content (BMC) and bone mineral density (BMD) with ageing and in relation to sex. In fact, in older people the mandibular BMC increases, albeit slightly, in males, while it decreases in females (3). This is explained by the presence in elderly males of a compensatory mechanism by which the inner cortical bone is thicker in order to maintain the stability of the atrophic mandibular body, with a reduction of the trabecular bone area. This mechanism does not seem to exist in postmenopausal women, because of OP and/or other such as hormonal and genetic systemic factors.

In addition, whenever teeth are lost the resorption of alveolar residual ridges progressively occurs whether the subject remains edentulous or is rehabilitated with removable prostheses. It has been also found that in patients with osteopenia or OP the porosity observed in the jaws (atrophy from disuse) increases, and that the improvement in chewing produced by prosthetic rehabilitation reduces the amount of bone resorption.

The correlation between changes in systemic BMD and the jawbones, has been assessed by Dual Photon Absorptiometry (DPA) Dual Energy X-ray Absorptiometry (DEXA) and Quantitative Computed Tomography (QCT) (4-5), suitably modified to determine the in vivo BMC. Recently, in some clinical studies the traditional techniques of panoramic radiograph of dental arches or intraoral (periapical or bitewing) radiograms have been used to assess bone density of the jaws. Most studies showed that these radiographic investigations, used on a routine basis by dentists, have the potential to raise suspicion for OP ( Table 2). The relevance of these findings for both early diagnosis of OP is straightforward and underlines the need, for both physicians and dentists, to familiarize with them.

**Table 2:**
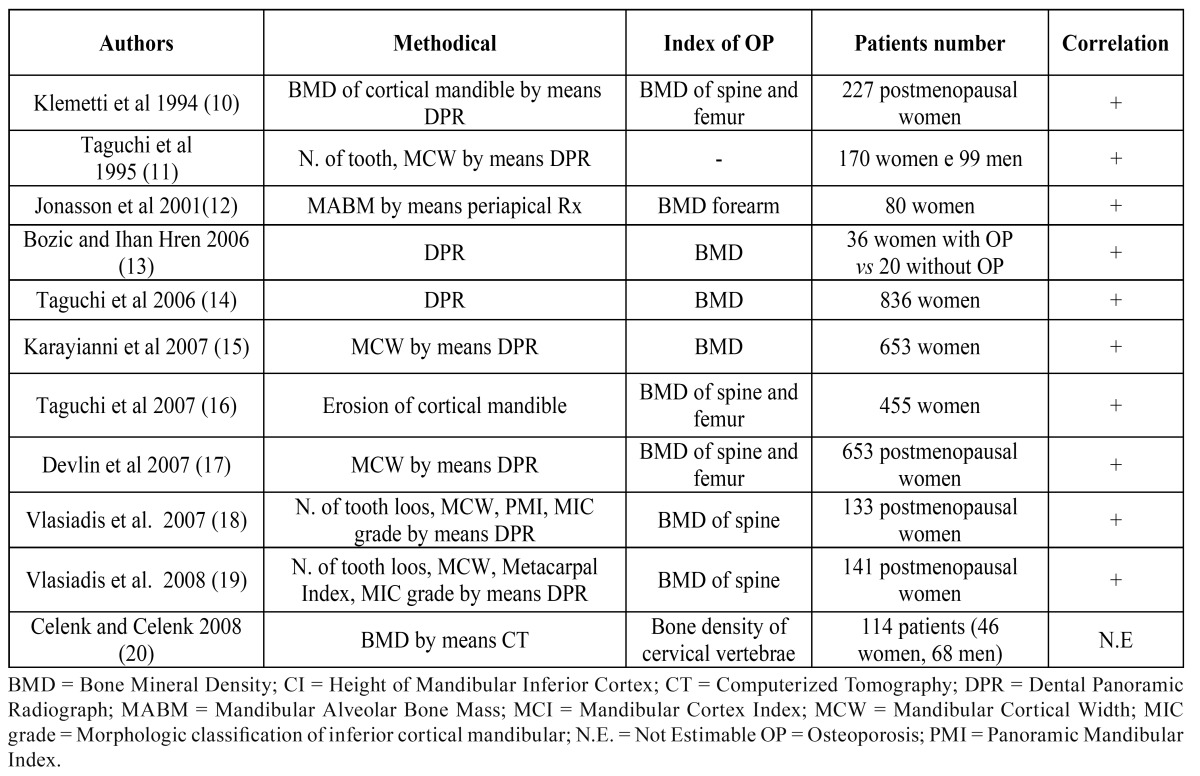
Assessment of oral BMD by means oral radiographic techniques.

-Periodontitis and Osteoporosis

PD is a complex disease entity with a multifactorial etiology in which the inflammatory response of the periodontal tissue to bacterial infection ends up with periodontal ligament detachment from the cement, formation of periodontal pockets, alveolar bone resorption, gingival recession, tooth mobility/migration.

Periodontitis is the major cause of alveolar bone resorption and tooth attachment loss resulting in tooth loss and, consequently, additional bone resorption. It is influenced by environmental factors as well as by genetic factors; thus, periodontal diagnosis requires oral/periodontal and assessment that`s to say: 1) patient`s medical and dental histories; 2) presence of clinical signs of inflammation of gingival tissues, including bleeding on probing; 3) pro-bing depths; 4); extent and pattern of attachment loss and bone defects 5) presence of various signs and symptoms, including pain, tooth mobility and amount of the observable plaque and calculus (6-7). Obtaining these data needs a thorough clinical and radiographic examination of both intraoral and extra oral structures (8).

The first reports on the possible association between systemic osteoporotic bone loss and local oral bone loss were released back in the`60s by Groen et al. (9) who suggested a possible correlation between the two conditions in patients presenting with both PD and BMD reduction at the forearm and at the spine. Since then, several studies have suggested a possible correlation between loss of systemic bone mass (osteopenia/osteoporosis) and loss of alveolar bone; the latter, however, has been addressed considering the following different series of parameters, many of which are only surrogate measures of PD.

a) clinical attachment level (CAL), depth of the periodontal pocket (PPD).

b) alveolar crest height (ACH) or height of the residual alveolar bone (ABH).

c) tooth loss (TL).

The most significant published works for each of them are detailed ( Table 3, Table 4).

**Table 3:**
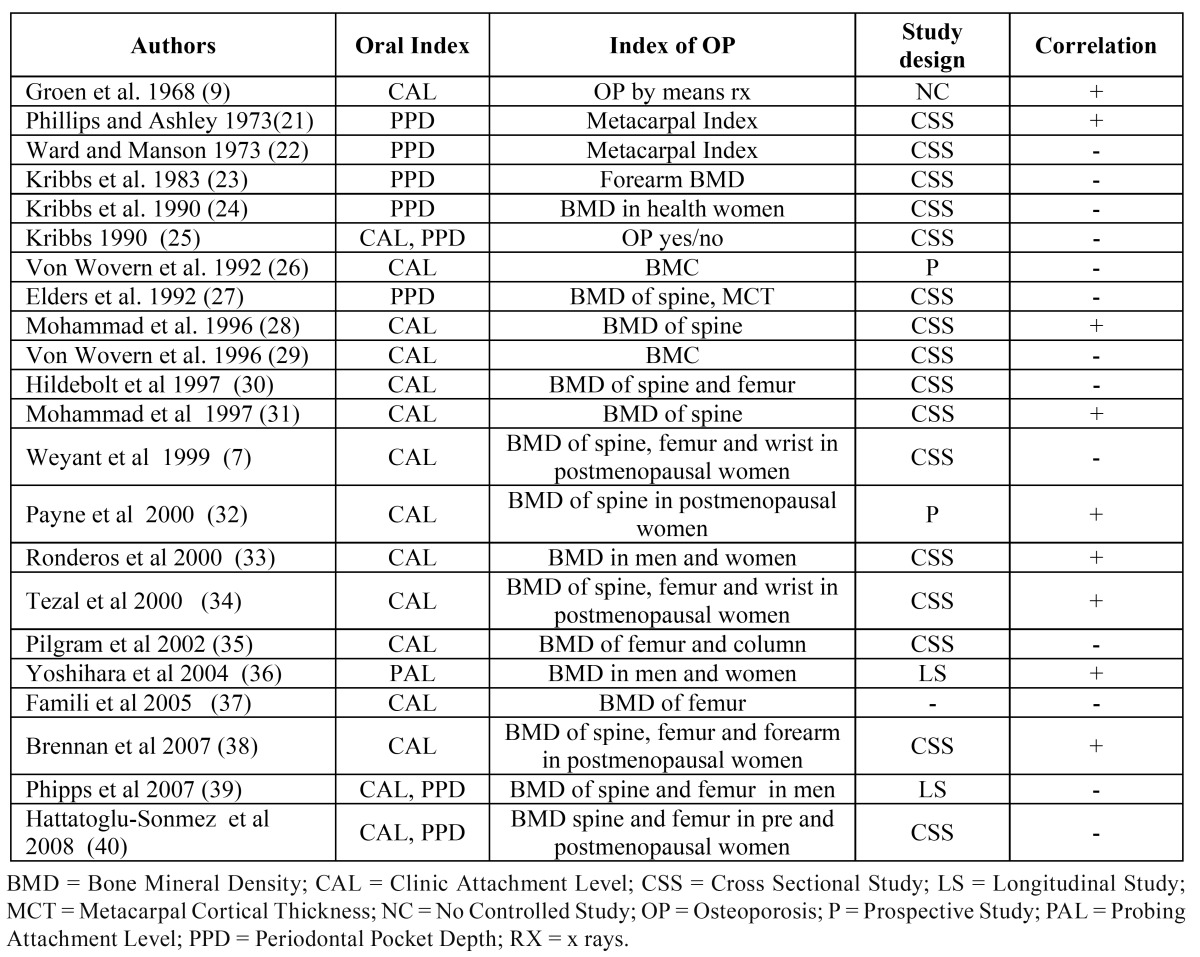
Studies on the association between indexes of OP and CAL/PPD.

**Table 4:**
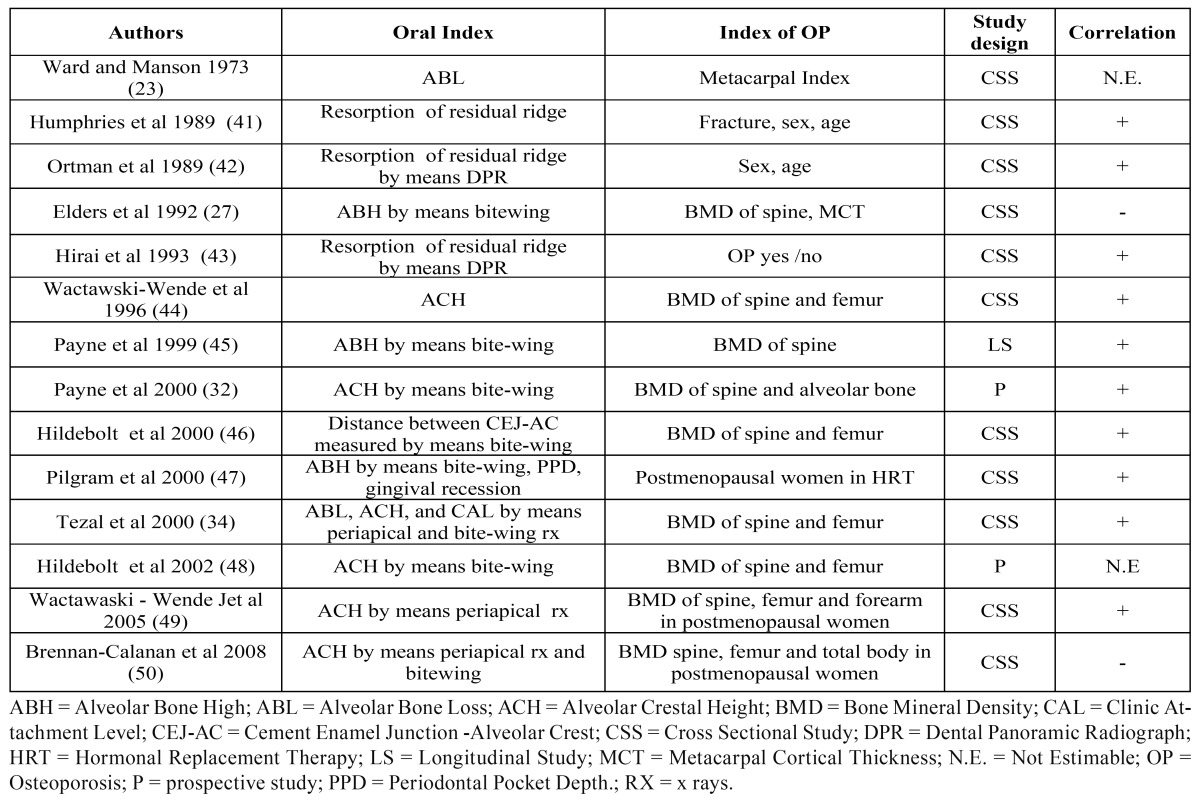
Studies on the association between BMD and ACH/ABH.

## Discussion

OP may affect jawbones and the resulting modifications visible at the routine radiological examination (i.e. dental panoramic radiograph) may be useful for OP early diagnosis. In addition, these modifications might potentially speed up periodontal tissues breakdown caused by PD (2). Under this point of view, although several reports on an epidemiologic basis, support a potential association between PD and OP, the comprehensive ana-lysis of the reported data provides conflicting results; however, it should be noted that reported studies have a wide variation in terms parameters used for assessing both OP and PD, thus a reliable comparison is somewhat problematic, and, in addition, their design (most of them are cross-sectional, uncontrolled and with small sample size restricted to postmenopausal women) is not adequate to draw robust conclusions (3-4).

On the other hand, besides the presence of common risk factors, a possible interplay between OP and PD is also suggested at a pathogenetic level. In fact, a bi-directional interference between PD and OP has been proposed: in particular, the reduced BMD, characterizing OP and the related alteration of trabecular pattern may lead to a more rapid jawbones resorption caused by PD, resulting in the invasion of periodontal bacteria (6-9). Invading bacteria, in turn, may alter the normal homeostasis of bone tissue, increasing osteoclastic activity and reducing local and systemic bone density by both direct effects (release of toxins) and/or indirect mechanisms (release of inflammatory mediators; in particular, interleukin-1 and interleukin-6) .

Thus, a relationship between OP and PD might be probable, but further prospective and sensitive studies are required in order to provide definitive evidence.

By now, available data underline the primary importance of dentists in the early diagnosis of OP, because of the opportunity to assess the health of the entire skeleton of the patient through dental radiography. This is of considerable clinical interest, considering that such dental radiological investigations are routinely performed for diagnosis and treatment of dental and periodontal diseases, which are particularly frequent in the same population affected by OP. This may also provide clues for new preventive strategies and/or early therapeutic approach resulting in a potential reduction of bone resorption and contributing to maintain bone biomechanical characteristics (e.g. architecture, remodelling, quality of matrix collagen and its mineralization).

In fact, the prevention of OP is the most rational and modern approach to defeat the disease (1), and early diagnosis is one of the foundations of modern medicine; the dentist seems to have an important role not only in monitoring/maintaining the oral and periodontal health and its relationships with systemic health, including OP, but also in drafting diagnostic/therapeutic paths and participating in counselling for OP in collaboration with general practioners and other specialists.
